# Identification of a Membrane-bound Prepore Species Clarifies the Lytic Mechanism of Actinoporins * ^♦^

**DOI:** 10.1074/jbc.M116.734053

**Published:** 2016-07-21

**Authors:** Koldo Morante, Augusto Bellomio, David Gil-Cartón, Lorena Redondo-Morata, Jesús Sot, Simon Scheuring, Mikel Valle, Juan Manuel González-Mañas, Kouhei Tsumoto, Jose M. M. Caaveiro

**Affiliations:** From the ‡ Department of Bioengineering, Graduate School of Engineering, University of Tokyo, Bunkyo-ku, Tokyo 113-8656, Japan,; the § Department of Biochemistry and Molecular Biology and; ¶ Biofisika Institute (UPV/EHU, CSIC), University of the Basque Country, P. O. Box 644, 48080 Bilbao, Spain,; the ‖ Structural Biology Unit, Center for Cooperative Research in Biosciences, CICbiogune, 48160 Derio, Spain,; the ** U1006 INSERM, Aix-Marseille Université, Parc Scientifique et Technologique de Luminy, 163 Avenue de Luminy, 13009 Marseille, France, and; the ‡‡ Institute of Medical Science, University of Tokyo, Minato-ku, 108-8639 Tokyo, Japan

**Keywords:** atomic force microscopy (AFM), cryo-electron microscopy, lipid vesicle, lipid-protein interaction, oligomerization, protein structure, cytolysin, pore forming protein

## Abstract

Pore-forming toxins (PFTs) are cytolytic proteins belonging to the molecular warfare apparatus of living organisms. The assembly of the functional transmembrane pore requires several intermediate steps ranging from a water-soluble monomeric species to the multimeric ensemble inserted in the cell membrane. The non-lytic oligomeric intermediate known as prepore plays an essential role in the mechanism of insertion of the class of β-PFTs. However, in the class of α-PFTs, like the actinoporins produced by sea anemones, evidence of membrane-bound prepores is still lacking. We have employed single-particle cryo-electron microscopy (cryo-EM) and atomic force microscopy to identify, for the first time, a prepore species of the actinoporin fragaceatoxin C bound to lipid vesicles. The size of the prepore coincides with that of the functional pore, except for the transmembrane region, which is absent in the prepore. Biochemical assays indicated that, in the prepore species, the N terminus is not inserted in the bilayer but is exposed to the aqueous solution. Our study reveals the structure of the prepore in actinoporins and highlights the role of structural intermediates for the formation of cytolytic pores by an α-PFT.

## Introduction

Pore-forming toxins (PFTs) ^5^ are proteins for defense and attack purposes (1, 2). These proteins function by opening pores across the cell membranes, triggering processes conducive to cell death (3). PFTs are commonly classified into α- and β-types according to the secondary structure of the transmembrane region of the pore (4–6). The classical route of pore formation begins with the interaction of the water-soluble monomer with the outer leaflet of the cell membrane, followed by the in-plane oligomerization of toxin subunits and self-assembly of a lytic transmembrane pore (7). Among the intermediate species that populate this pathway, prepore particles bound to lipid bilayers have been described in the class of β-PFTs (8–12). In the class of α-PFTs, prepore structures have been proposed for cytolysin A from *Escherichia coli* (6, 13, 14), although its direct visualization still remains elusive.

In actinoporins, a class of α-PFTs secreted by sea anemones, it is far less clear if a stable prepore is assembled before formation of the lytic transmembrane pore. Structurally, actinoporins are composed of a rigid β-sandwich core (mediating binding to the membrane) and two flanking α-helices, as shown for the actinoporin FraC (20 kDa, 179 residues) (15). The N-terminal region of FraC is made of an amphipathic α-helix and neighboring residues that insert collectively in the bilayer and, together with structural lipids from the membrane, line the lytic pore (15). This remarkable metamorphosis is fully reversible under certain environmental conditions (16).

**FIGURE 1.**
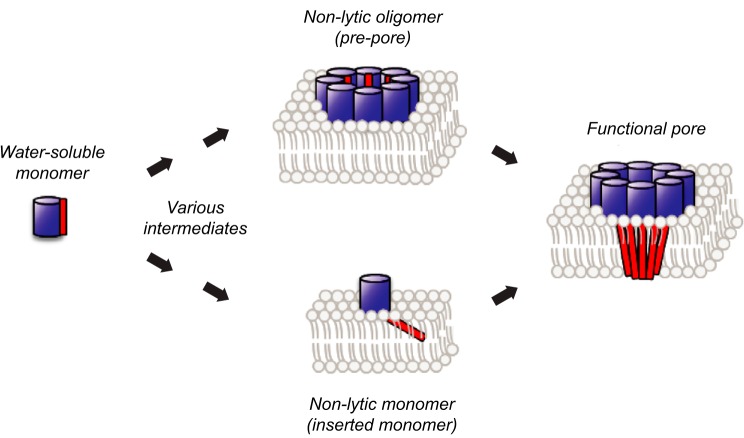
**Two alternative routes for pore formation in actinoporins.** The binding of the water-soluble monomer to the cell or model membranes leads to a lytic (active) pore by at least two alternative routes, as shown in the figure. *Top,* formation of a non-lytic oligomer (prepore) precedes insertion into the membrane (20). *Bottom,* insertion of the N-terminal region into the membrane occurs prior to oligomerization of the functional pore (21).

A variety of membrane-bound species has been proposed in the mechanism of actinoporins (17–19). However, the nature of some of the intermediate species and the order at which they appear during pore formation remain unclear. Some studies have proposed that the protein subunits first assemble into an oligomeric prepore, followed by the concerted insertion of the N-terminal region in the lipid bilayer that gives rise to the functional pore (20). Other studies, on the contrary, have suggested that the α-helix inserts deeply in the membrane prior to the oligomerization step, and therefore, this model does not contemplate the appearance of stable prepores (Fig. 1) (21).

Here, we have visualized a non-lytic oligomer of FraC bound to large unilamellar vesicles (LUVs) by using cryo-EM and bound to supported planar bilayers by employing AFM. The dimensions of the cryo-EM model and the high resolution AFM images indicate that the prepore is made of eight protein subunits, a result consistent with the oligomerization number of the active pore (15). Biochemical assays indicate that, in the prepore species, the first few residues of the N terminus are not embedded in the lipid phase but instead are exposed to the aqueous environment. Our results reinforce the idea that protein oligomerization occurs prior to the complete insertion of the N-terminal region into the membrane, thus clarifying a critical aspect of the lytic mechanism of actinoporins.

## Experimental Procedures

### 

#### 

##### Materials

Sphingomyelin (SM) from porcine brain and chicken egg, 1,2-dilauroyl-*sn-*glycero-3-phosphocholine (DLPC), 1,2-dipalmitoyl-*sn*-glycero-3-phosphocholine (DPPC), and 1,2-dioleoyl-*sn*-glycero-3-phosphocholine (DOPC) were from Avanti Polar Lipids. 8-Aminonaphthalene-1,3,6-trisulfonic acid (ANTS), *p*-xylene-bis-pyridinium bromide, 1,1′-dioctadecyl-3,3,3′,3′-tetramethylindocarbocyanine perchlorate (DiIC_18_), and Alexa Fluor 633 succinimidyl ester were from Thermo Fisher Scientific. Proteinase K (PK) was purchased from Sigma.

##### Protein Expression and Purification

Expression and purification of FraC were carried out as described previously (22). Briefly, FraC expression was induced in *E. coli* BL21 (DE3) cells and then purified to homogeneity by ion-exchange and size-exclusion chromatography. Oxidation of the double-cysteine mutein was carried out as described by Hong *et al.* (23).

##### Protein Labeling

To visualize FraC by fluorescence microscopy, the protein was labeled with the amine-reactive fluorescent dye Alexa Fluor 633 succinimidyl ester. The succinimidyl ester moiety reacts with non-protonated aliphatic primary amine groups of the protein. The protein-dye conjugate was prepared following the instructions supplied by the manufacturer. Briefly, 740 μl of FraC at 180 μm in a buffer of 90 mm bicarbonate (pH 8.3) were mixed with 45 μl of fluorescent dye previously dissolved in DMSO. The mixture was incubated for 1 h at room temperature with constant stirring. To stop the reaction, 80 μl of freshly prepared hydroxylamine (1.5 m) was added and incubated for 1 h at room temperature. Unreacted reagent was separated from the conjugate by elution of the reaction mix through a Sephadex G-15 (GE Healthcare) packed column. The protein conjugate was tested for activity using surface pressure measurements and hemolysis assays, showing a similar behavior to that of the unlabeled protein (data not shown), and thus ruling out detrimental effects caused by the dye.

##### Liposome Preparation and Leakage Assays

LUVs of 100 nm were formed by extrusion as described previously (24). The lipid concentration was determined according to Bartlett (25). For the leakage assays, four different populations of LUVs made of DLPC, DPPC, DOPC, and SM/DOPC (1:1) were prepared as described above in a buffer containing 10 mm HEPES (pH 7.5), 50 mm NaCl, 25 mm ANTS (the fluorescent probe), and 90 mm *p*-xylene-bis-pyridinium (the quencher), followed by washing the liposomes with isosmotic buffer composed of 10 mm HEPES and 200 mm NaCl (pH 7.5) in a PD-10 column (GE Healthcare). Leakage of encapsulated solutes was assayed as described by Ellens *et al.* (26). Briefly, LUVs were incubated with FraC at room temperature for 30 min to ensure, as much as possible, completion of vesicle lysis at each protein concentration employed. Release of encapsulated solutes to the external medium dilutes the quencher and fluorophore (ANTS), resulting in an increase of the emission of fluorescence. The fluorescence was measured in a PHERAstar Plus microplate reader (BMG LABTECH, Ortenberg, Germany) with excitation/emission wavelengths of 350/520 nm. Complete release of the ANTS was achieved by solubilization of the liposomes with Triton X-100 (0.1% w/v). The percentage of leakage was calculated as shown in Equation 1,

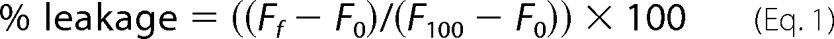
 where *F_f_* is the fluorescence measured after addition of the toxin; *F*_0_ is the initial fluorescence of the liposome suspension, and *F*_100_ is the fluorescence after addition of detergent.

##### Surface Pressure Measurements

Surface pressure measurements on lipid monolayers made of DLPC, DPPC, or DOPC were carried out in a Micro-Trough-S instrument (Kibron, Finland) at room temperature with constant stirring. In these experiments, the lipid was applied on the air-water interface to the desired initial surface pressure. The protein (1 μm) was injected in the aqueous subphase and the change of surface pressure recorded. The maximum surface pressure (π_max_) was determined with Equation 2,

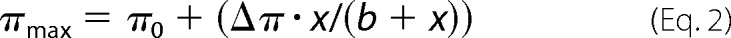
 where π_0_ is the initial surface pressure; Δπ is the change in surface pressure; *x* is time; and *b* is the time necessary to reach half-effect (Δπ/2) (27). The critical pressure (π*_c_*) corresponds to the initial surface pressure of the lipid monolayer at which the protein no longer penetrates the surface, calculated by least squares fitting at Δπ = 0.

##### Cryo-EM of FraC Inserted in Model Membranes

LUVs composed of DOPC were incubated with FraC (5 μm) at a protein/lipid ratio of 1:160 for 30 min. Holey-carbon grids were prepared following standard procedures and were observed in a JEM-2200FS/CR transmission electron microscope (JEOL Europe, Croissy-sur-Seine, France) operated at 200 kV at liquid nitrogen temperature. A set of 1,562 individual pore particles were manually selected and recorded on a CCD camera under low dose conditions at ×60,000 magnification resulting in a final pixel size of 1.72 Å. An in-column ω energy filter was used to improve the signal to noise ratio of the images.

The images were corrected for the contrast transfer function by flipping phases after estimation of parameters in EMAN (28). The two-dimensional images were classified by maximum likelihood and hierarchical clustering procedures within the XMIPP software package (29). The starting three-dimensional model was generated using reference-free alignment, classification, and common-lines procedures implemented in EMAN. This was followed by iterative refinement using a projection matching scheme in SPIDER package (30).

The rigid-body fitting was performed by maximization of the sum of map values at atom positions and by improvement in the coefficient of correlation between simulated maps from the atomic structures and the cryo-EM density map in Chimera (31). The correlation between the atomic structure of FraC and cryo-EM map suggested a resolution of ∼30 Å.

##### Preparation of Giant Unilamellar Vesicles (GUVs) for Confocal Fluorescence Microscopy

GUVs made of DOPC/DPPC (20:80) were prepared by electroswelling on a pair of platinum wires as described previously in a temperature-controlled chamber (32). Briefly, a mixture of 0.2 μg/μl lipid and 0.2% DiIC_18_ was applied on the chamber and dried under vacuum. The sample was then hydrated with 10 mm HEPES, 200 mm NaCl (pH 7.5) at 61 °C. This temperature was selected to prevent lipid demixing. To form the vesicles, current was applied in three consecutive steps under AC field conditions and a sinusoidal wave function as follows: (i) 500 Hz, 0.22 V (35 V/m) for 6 min; (ii) 500 Hz, 1.9 V (313 V/m) for 20 min; and (iii) 500 Hz, 5.3 V (870 V/m) for 90 min. After vesicle formation was completed, the chamber cooled down passively to room temperature.

##### Inverted Confocal Fluorescence Microscopy

For the visualization of GUVs and labeled protein, the chamber containing the GUVs was placed on a D-Ellipse C1 inverted confocal fluorescence microscope (Nikon, Melville, NY) at room temperature. The excitation wavelengths were 561 nm (for DiIC_18_) and 633 nm (for Alexa Fluor 633). The fluorescence signal was collected into two different channels with bandpass filters of 593/40 and a 650-nm long pass. The objective used was a ×60 oil immersion with NA of 1.45. Image treatment was performed with the EZ-C1 3.20 FreeViewer software.

##### Preparation of GUVs for AFM

GUVs made of SM/DOPC (1:1) were prepared by the electroswelling technique as described previously (33). A volume of 30 μl of 1 mg/ml lipids dissolved in chloroform/methanol (3:1) was deposited in two glass plates coated with indium tin oxide (70–100 ohm resistivity, Sigma) and placed in the desiccators for at least 120 min for complete solvent evaporation. A U-shaped rubber piece of ∼1-mm thickness was sandwiched between the two indium tin oxide side slides. This chamber was filled with ∼400 μl of 200 mm sucrose and was exposed to 1.2 V AC current (12 Hz sinusoidal for 2 h, 5 Hz squared for 10 min). The resulting suspension was collected in a vial and used within several days.

##### Supported Lipid Bilayer Preparation for AFM

A total of 1 μl of a suspension of GUVs was deposited onto freshly cleaved 1-mm^2^ mica pretreated with 1 μl of 10 mm Tris-HCl, 150 mm KCl (pH 7.4) (imaging buffer), and incubated for 15 min at room temperature. The resulting supported lipid bilayers were carefully rinsed with imaging buffer before image collection and always kept under an aqueous environment. During imaging, FraC toxin was injected into the fluid cell to give a final concentration of about 10 μm.

##### AFM Imaging

AFM was performed at room temperature on a high speed AFM 1.0 instrument (RIBM, Japan) equipped with short high speed AFM cantilevers (∼8 μm, NanoWorld, Switzerland) with nominal resonance frequency of ∼1.2 and ∼0.7 MHz in air and liquid, respectively, and a nominal spring constant of ∼0.15 Nm^−1^. Image acquisition was operated using optimized feedback by a dynamic PID controller. Small oscillation free (*A*_free_) and set point (*A*_set_) amplitudes of about 1 and 0.9 nm, respectively, were employed to achieve minimum tip-sample interaction. Typically, pixel sampling ranges from 100 × 100 pixels and 200 × 200 pixels and frame rate between 500 and 800 ms per frame.

##### AFM Data Analysis

AFM data were analyzed in ImageJ and with self-written image analysis scripts (movie acquisition piezo drift correction) in ImageJ (34). To obtain the high resolution images shown in Figs. 5 and 6, five consecutive frames were time-averaged. Histogram distributions were analyzed with Igor and Origin.

##### Protease Susceptibility Assay

PK (50 μm) was incubated with FraC (50 μm) for 24 h at room temperature in 50 mm Tris, 200 mm NaCl, 5 mm CaCl_2_ at pH 7.4. In the assays with lipids, FraC was incubated with LUVs (7.5 mm) made of DOPC or SM/DOPC (1:1) for 30 min prior to the addition of PK. The reaction was stopped by adding phenylmethylsulfonyl fluoride at a final concentration of 5 mm for 10 min and analyzed by SDS-PAGE.

##### N-terminal Sequencing

SDS-PAGE protein bands after the reaction with PK were transferred to a polyvinylidene fluoride membrane (Bio-Rad) and stained with Ponceau 3R solution for 1 h to verify the transfer was successful. Excess dye was removed by successive rinses with water. The bands of protein were excised from the membrane, and their N termini were sequenced by standard techniques (35).

## Results

### 

#### 

##### Interaction of FraC with PC Membranes

Because actinoporins are specifically activated by membranes containing the lipid SM, the use of lipid compositions in which SM is absent but where the toxin maintains a strong interaction with the vesicles may reveal structural intermediates not detectable by other means. We first evaluated the interaction of FraC with vesicles made of various types of the lipid PC (a lipid displaying the same phosphocholine headgroup moiety as that of SM) to determine the optimum PC species yielding the highest possible association between protein and liposomes.

**FIGURE 2.**
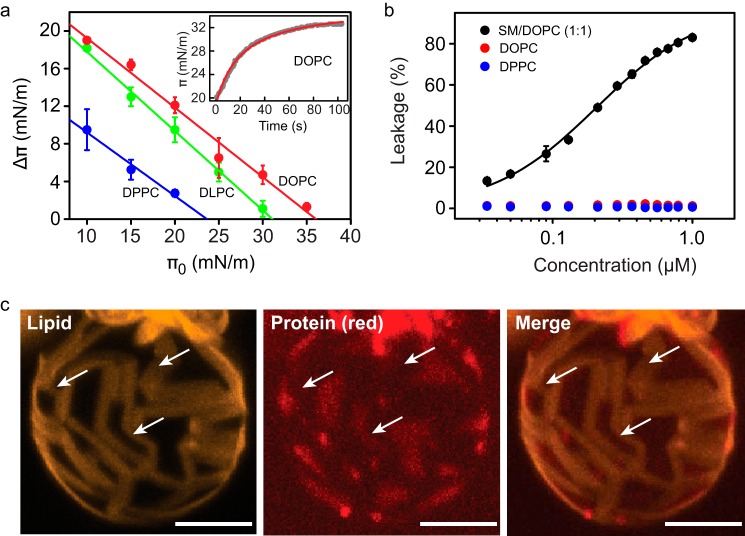
**Interaction of FraC with model membranes.** *a,* change in surface pressure of lipid monolayers composed of DOPC (*red*), DLPC (*green*), or DPPC (*blue*) after treatment with FraC (1 μm). The parameter π*_c_* corresponds to the value of π_0_ where the regression line intersects the *abscissa*. The *inset* shows a representative example of the kinetic profile of insertion of FraC in DOPC monolayers (π_0_ = 20 mN/m, *gray trace*). A hyperbola (*red line*) was fitted to the experimental data, from which the value of Δπ was determined. *b,* lack of lytic activity of FraC in LUVs made of DOPC (*red*) or DPPC (*blue*). The data obtained with SM/DOPC (1:1) represent a positive control (*black*). The LUVs made of DLPC are permeable to encapsulated dyes in the absence of protein (spontaneous leakage), and thus the data obtained with them was not considered. For the experiments in *a* and *b,* the mean and standard deviation of three independent measurements was plotted. *c,* binding of FraC to GUVs made of DOPC/DPPC (20:80) supplemented with 0.2% DiIC_18_. This probe partitions in the ordered phase regions (*yellow* domains) (52, 53). Protein (*red*) was added to a final concentration of 1.3 μm. Lipid and protein were visualized with a 593/40-nm bandpass filter (*yellow, left panel*) or with a 650-nm long pass filter (*center panel*), respectively. Merged images are shown on the *right panel*. The *white arrows* point at liquid disordered regions (*dark* domains) where FraC is preferentially located. The *white scale bar,* 5 μm.

The three PC lipid species examined were DLPC, DPPC, and DOPC each differing in the length and degree of saturation of their acyl chains. To evaluate the degree of interaction of FraC with these lipids, we measured the magnitude of the insertion of the protein in a monolayer of lipid molecules at the water-lipid interface (Fig. 2*A*). We determined the surface pressure at which the protein will no longer penetrate, known as critical pressure (π*_c_*) (27). The lipid composition at which π*_c_* was highest corresponded to that of monolayers composed of DOPC (π*_c_* = 36.1 ± 1.6 mN/m) followed by that of monolayers made of DLPC (π*_c_* = 31.1 ± 1.3 mN/m). The insertion of FraC in DPPC monolayers was meager (23.6 ± 1.0 mN/m). These data suggest that the toxin associates more readily with lipids in the liquid-expanded phase such as DOPC and DLPC than those in the liquid-condensed phase (DPPC) (36–38). However, the protein does not generate pores in LUVs when SM was absent regardless of the lipid phase (Fig. 2*B*). A close association between actinoporins and lipid monolayers thus does not guarantee effective formation of pores in a lipid bilayer system as there are other physicochemical properties involved in pore formation, such as lipid phase coexistence and the presence of SM (39).

Additional evidence describing the lipid preference of FraC was gathered by visualizing the binding of the fluorescently labeled toxin to GUVs composed of DOPC/DPPC (20:80). In these experiments, the fluorescent dye conjugated to the toxin colocalized with the DOPC domains (*dark areas* in Fig. 2*C*) indicating that the toxin preferentially binds to the fluid phase domains than to the gel domains, a result consistent with the observations made with monolayers of FraC and leakage assays of sticholysin II (40). Based on these results, membranes composed of DOPC were selected for structural studies analyzing the conformation of FraC bound to membranes in a non-lytic scenario.

##### Cryo-EM

**FIGURE 3.**
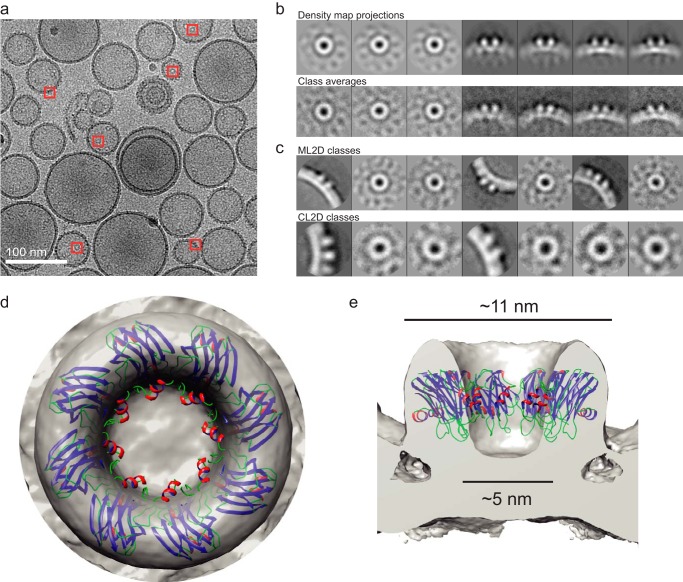
**Structure of the oligomeric prepore of FraC bound to DOPC vesicles.** *a,* representative image of FraC in DOPC vesicles obtained by cryo-EM. Top and side views of the protein oligomers were selected (*red squares*) for subsequent classification analysis. The *scale bar* corresponds to 100 nm. *b,* density map projections (*top row*) and two-dimensional class-averaged particles (*bottom row*) employed to build a three-dimensional model of the protein oligomer (see below). *c,* set of particles obtained by maximum-likelihood (*ML2D*, *top row*) and hierarchical clustering (*CL2D*, *bottom row*) procedures. *d,* top view, and *e,* side view of the three-dimensional model of the prepore of FraC bound to vesicles of DOPC. The atomic model of FraC was built as an octamer using the coordinates of the protomer of FraC prior to pore formation (entry code 4TSL).

To visualize the structure of membrane-bound FraC, vitrified samples of toxin-treated DOPC liposomes were imaged by cryo-EM. Ring-shaped particles covering the lipid vesicles were attributed to protein oligomers (Fig. 3*A*). A total of 1,562 top view and side view images were selected to build a three-dimensional model of the protein oligomer. The model was built by common-lines procedures, followed by iterative refinement using projection matching of the class-averaged images and the density map projections (Fig. 3*B*). A second classification method based on maximum likelihood (41) and hierarchical clustering approaches (Fig. 3*C*) (42) rendered images similar to those used to generate the final density map.

The reconstructed image consisted of a doughnut-shaped ring with an external and internal diameter of ∼11 and ∼ 5 nm, respectively (Fig. 3, *D* and *E*). These dimensions are very similar to those of the crystallized pore in the active state (15). However, unlike the cryo-EM model of the pore bound to SM/DOPC (1:1) liposomes (20), the oligomer bound to DOPC vesicles does not span the lipid membrane (see below). This architecture is consistent with a non-lytic oligomeric species resembling a prepore. A rigid-body fitting of an octameric model of FraC based on the atomic structure of the transmembrane pore of FraC achieved a high cross-correlation coefficient (cc = 0.82, Fig. 3, *D* and *E*) (15). The oligomeric model fits well within the perimeter of the cryo-EM map, except for the N-terminal region, which lies outside the electron density map suggesting that it is either resting on the surface of the membrane or inserted in the hydrophobic core of the membrane (21, 23, 43). A nonamer of FraC mimicking the structure of a crystallized oligomer of FraC in the presence of detergents (20, 44) was also fitted in the cryo-EM maps, yielding a cross-correlation coefficient only slightly worse (cc = 0.81) than that of the octamer. The fitted nonamer displayed a few clashes between protomers, in contrast to an oligomer made of 10 subunits in which the numerous collisions between protein chains made the decamer prepore unfit for the electron density (data not shown). From these data we cannot rule out the existence of a minor population of nonameric prepore species preceding a hypothetical nonameric pore, as discussed previously (15).

**FIGURE 4.**
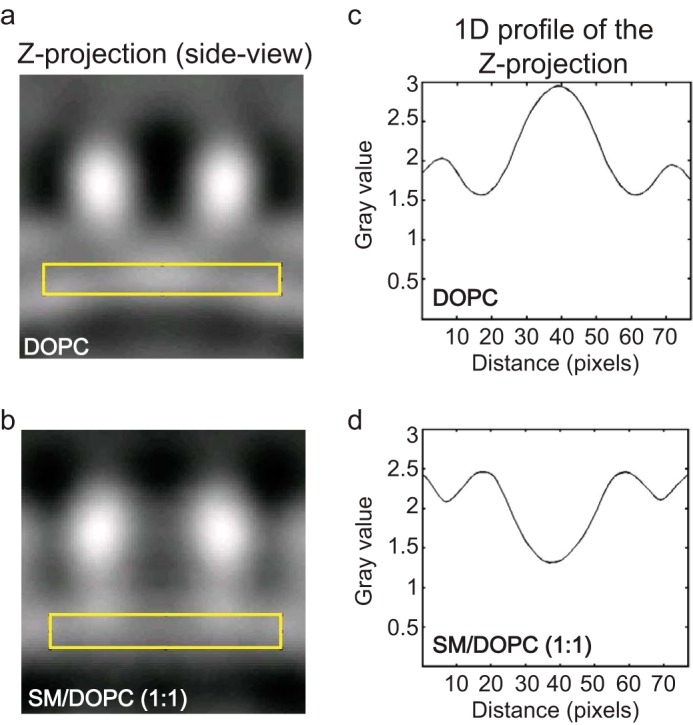
**Electron density of FraC bound to vesicles.** Side view (Z-projection) of oligomers of FraC bound to vesicles of DOPC (*a*) or SM/DOPC (1:1) (*b*). The *yellow rectangle* indicates the region where the one-dimensional profile of the Z-projection (shown in *c* and *d*) was calculated. The intensity of the electron density is expressed in gray values. *b* and *d* correspond to the analysis carried out with published data (20), although we note that the analysis presented here has not been shown elsewhere.

A comparative analysis of the electron density distribution along the central section of the oligomer of FraC in DOPC membranes and in SM/DOPC (1:1) clearly shows that the differences between pores and prepores occur in the critical transmembrane region. To perform this comparison, we employed the previously reported cryo-EM map of the active pore (20). For the analysis, three-dimensional volumes focused on the pore regions (shown within *yellow rectangles* in Fig. 4, *A* and *B*) were projected into two-dimensional images, and the gray values of the images (resulting from the accumulation of three-dimensional density values) were plotted in one-dimensional profiles (Fig. 4, *C* and *D*). The structure of FraC in DOPC membranes (Fig. 4*A*) reveals a peak of high density values in a central lobe at the membrane level below the vestibule of the oligomer (Fig. 4*C*), whereas in SM/DOPC (1:1) membranes (Fig. 4*B*), the same region is characterized by low density values (Fig. 4*D*). These features are consistent with the absence (in DOPC membranes) or the presence (in SM/DOPC (1:1) membranes) of a transmembrane pore. In the former, the high density central region likely represents the accumulation of membrane lipids and N-terminal α-helices detached from the β-core of the toxin. In contrast, in membranes containing SM, the central region of the oligomer displays lower electron density because the α-helices span the membrane, and consequently the lipids are cleared off producing an aqueous pore.

##### AFM

**FIGURE 5.**
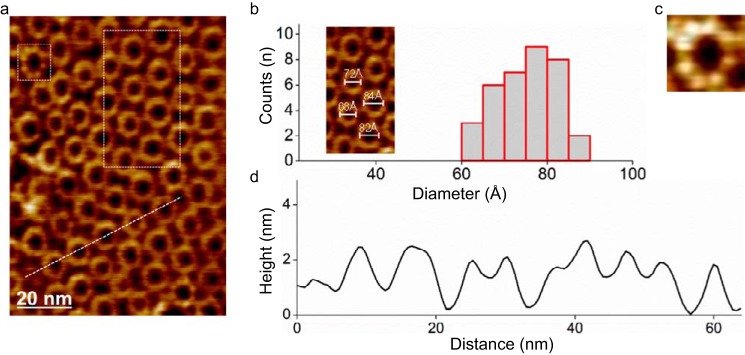
**Visualization of pores of WT FraC with AFM.** *a,* two-dimensional packing of ring-shaped oligomers of WT FraC on supported lipid bilayers composed of the lipid mixture SM/DOPC (1:1). *b,* diameter distribution analysis (peak-to-peak distances of the protein protrusion in the height profile). The average diameter of the particles was 75 ± 6 Å (mean ± S.D. from the Gaussian distributions). *Inset,* detail of the particles inside the *white dashed rectangle* in *a. c,* magnification (13-nm frame size) of a single FraC oligomer in *a* (*white dashed square*). *d,* cross-section profile (*left to right*) of FraC oligomers shown in *a* (*white dashed line*). The molecules are packed with a center-to-center distance of ∼112 Å.

In the presence of supported lipid bilayers composed of the equimolar mixture SM/DOPC (1:1), WT FraC self-assembles in a dense array of closely packed oligomers as determined by AFM (Fig. 5). These oligomers, presumably corresponding to pore particles, cover the SM-rich domains in an arrangement previously observed in FraC and other actinoporins (44, 45) or the SM-specific PFT lysenin (46). The cross-section profile of the oligomeric complexes reveals an average diameter of 7.5 ± 0.6 nm, a value in good agreement with the mean diameter (average of outer and inner diameters) of the pore determined by x-ray crystallography (∼8 nm). Eight protein chains are observed in three well resolved pore particles encountered (see for example Fig. 5*C*).

**FIGURE 6.**
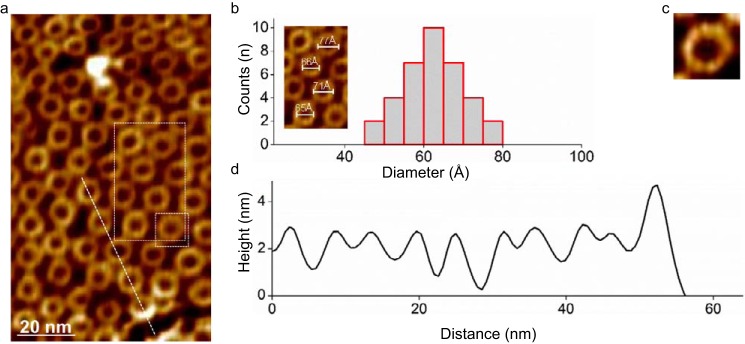
**Visualization of prepores of 8-69^OX^ FraC with AFM.** *a,* two-dimensional packing of ring-shaped oligomers of 8-69^OX^ FraC on supported lipid bilayers composed of the lipid mixture SM/DOPC (1:1). *b,* diameter distribution analysis (peak-to-peak distances of the protein protrusion in the height profile). The average diameter was 62 ± 7 Å (mean ± S.D. from the Gaussian distributions). *Inset,* detail of the particles inside the *white dashed rectangle* in *a*. The slightly smaller diameter compared with the WT suggests a tighter association of the subunits in the 8-69^OX^ FraC mutant. *c,* magnification (12-nm size frame) of a single prepore of FraC 8-69^OX^ (*white square* in *a*). *d,* cross-section profile (*left to right*) of prepore particles of FraC 8-69^OX^ (*white line* in *a*). The molecules packed with a center-to-center distance of ∼108 Å.

Because prepores of FraC were not resolved in DOPC (high diffusivity prevented AFM contouring), a construct of FraC bearing a double cysteine mutation (V8C/K69C, termed 8-69^OX^) was instead examined on supported membranes made of SM/DOPC (1:1). Under oxidizing conditions, the N-terminal segment of this mutein is covalently attached to the protein core by means of a disulfide bond, preventing the protein from generating a transmembrane pore, and thus inactivating the toxin (15, 23). As with WT FraC, the construct 8-69^OX^ also gave rise to a dense array of pore-like particles (Fig. 6), indicating that the protein readily oligomerizes in the presence of membranes even if the N-terminal region remains attached to the protein. The average diameter of these particles (6.2 ± 0.7 nm) is somehow smaller than that of WT protein, reflecting the influence of the N-terminal region attached to the β-core region. Because of the constraints imposed by the disulfide bond, the conformation of the N-terminal region in 8-69^OX^ is likely to differ from that of WT FraC bound to liposomes made of DOPC (Fig. 3, *D* and *E*). To further investigate this question, we employed biochemical assays (see below).

##### Protease Susceptibility of the Membrane-bound Toxin

It was shown that FraC bound to LUVs exhibits different susceptibility to PK depending on the lipid composition of the membrane (15). The incubation of FraC with PK generated a product of smaller size when the toxin was bound to DOPC vesicles as compared with those generated in the presence of SM/DOPC (1:1) vesicles (15), although the basis of this difference was not explained. In view of the new prepore oligomeric species described herein, we hypothesized that the N terminus of this prepore is located in a solvent-exposed environment accessible to PK, whereas in the pore the N terminus is deeply inserted in the membrane and thus inaccessible to the protease. To verify this hypothesis and determine the extent of the digestion, we incubated samples of FraC with PK followed by their separation by SDS-PAGE and N-terminal sequencing.

**FIGURE 7.**
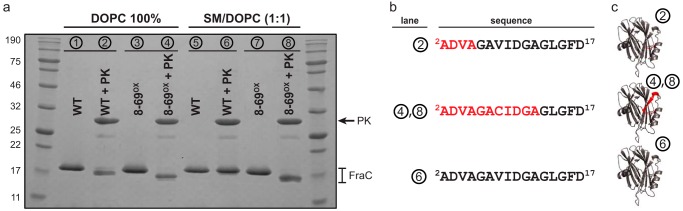
**Protection of FraC from PK in the presence of liposomes.** *a,* SDS-PAGE of the products obtained after the incubation of WT and 8-69^OX^ FraC with DOPC vesicles (*lanes 1–4*) or with SM/DOPC (1:1) (*lanes 5–8*) in the absence and in the presence of PK. *b,* N-terminal sequence of FraC after digestion with PK. The *circled numbers* correspond to the lane in the SDS-PAGE. Residues highlighted in *red* were digested by PK. The first 16 residues of the recombinant WT protein expressed in *E. coli* are ADVAGAVIDGAGLGFD (54). *c,* location of the residues digested by PK (in *red*) are depicted in the three-dimensional structure of the monomer of FraC (Protein Data Bank code 3VWI).

The incubation of PK with FraC in the presence of DOPC vesicles yields a fragment of smaller molecular weight than that of the untreated protein (shown in the 20-kDa region). In contrast, in the presence of SM/DOPC (1:1) vesicles, the bands of treated and untreated toxin display the same molecular mass (Fig. 7*A*). The mutein 8-69^OX^ bound to membranes was also employed, because its N terminus remains exposed to the solvent constrained by the disulfide bond. As expected, 8-69^OX^ was also susceptible to the proteolytic activity of PK in DOPC and SM/DOPC (1:1) vesicles. To determine the extent of the cleavage, the proteins were subjected to sequencing of their N-terminal regions. The sequencing data revealed that, in the presence of vesicles of DOPC, FraC WT and 8-69^OX^ were cleaved at the N terminus by PK, rendering products in which the first 4 and the first 11 residues, respectively, were missing (Fig. 7, *B* and *C*). FraC bound to SM/DOPC (1:1) was not digested by PK as expected from the position of the band in the SDS-polyacrylamide gel, whereas 8-69^OX^ was cleaved at the same position seen after incubation with vesicles of DOPC. These results demonstrate that the N terminus of FraC in DOPC vesicles (prepore configuration) is accessible to PK, *i.e.* this region is not embedded in the lipid bilayer.

## Discussion

The characterization of structural intermediates populating the assembly pathway of PFTs is a necessary endeavor to elucidate the mechanism of pore formation. Membrane-bound oligomeric structures poised for membrane disruption are commonly referred to as prepores and have been visualized in lipid bilayers only for β-PFTs. In contrast, the existence of prepores in α-PFTs is controversial. An example is the family of actinoporins, where a strong debate is held about the existence or not of these non-lytic oligomers (20, 21, 47). Until now, the evidence supporting a prepore in actinoporins was based on the crystal structure of a non-lytic nonameric ensemble solved for FraC (20).

Herein, we have described a low resolution membrane-bound oligomer consistent with the ability of FraC to assemble as a prepore on biological membranes. We employed a protein concentration above physiological levels to ensure a large and homogeneous population of prepore species bound to the liposomes, thus facilitating their visualization by cryo-EM and AFM. The relevance of the prepore structure obtained herein is demonstrated by the readiness of actinoporins to induce lysis in liposomes made of PC upon generation of lipid domains *in situ* (39). The size and stoichiometry of the prepore in the cryo-EM (Figs. 3 and 4) and AFM (Fig. 6) images were comparable with the crystallized pore species (15). The cryo-EM reconstruction map is not consistent with a transmembrane pore, an argument strengthened by the comparison side-by-side with cryo-EM data of pores of FraC embedded in vesicles of SM/DOPC (1:1) (20).

Electron density gradient analysis and protease digestion assays suggest a close association of the N-terminal region with the membranes in a position approximately parallel to the plane of the membrane as was described before for other actinoporins (43, 48). Evidence that this oligomer precedes pore formation is inferred from a previous study carried out with the actinoporin equinatoxin II. In that study it was shown that the addition of phospholipase C to vesicles of PC decorated with toxin promoted vesicle lysis by the *in situ* generation of lipid domains (39).

**FIGURE 8.**
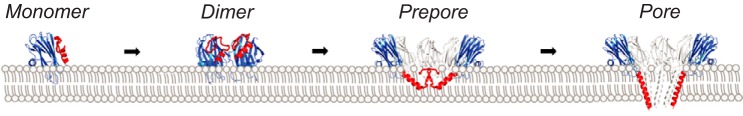
**Model for pore formation by FraC.** A toxin monomer binds the membrane. The membrane promotes protein-protein interactions between monomers to produce a dimer (15) leading to prepore upon successive addition of monomer and/or dimers to the growing oligomer. In the prepore, the N-terminal α-helices are partially embedded in the membrane with their N termini exposed to the aqueous phase. The conversion to the transmembrane pore would be achieved by the concerted penetration and elongation of the helices across the lipid bilayer. The structures of the monomer, dimer, and pore were retrieved from the Protein Data Bank codes 3VWI, 4TSL, and 4TSY, respectively. The prepore illustrates a tentative model consistent with the presented in this study.

Our results suggest a model where the N-terminal α-helices penetrate the bilayer in a concerted manner (Fig. 8), an alternative mechanism to that in which helix penetration occurs before protein oligomerization (21). Although pore formation by the successive insertion of single α-helices cannot be completely ruled out in membranes made of SM/DOPC (1:1), simple thermodynamic considerations suggest that would not be the case. The penetration of individual α-helices containing a large number of charged residues (FraC displays three Asp and one Glu in this region) in the hydrophobic core of biological membranes would be strongly disfavored (49–51).

In conclusion, our study clarifies the structure of a key intermediate, known as prepore, in the self-assembly pathway of actinoporins belonging to the class of α-PFTs. The characterization of the prepore in actinoporins highlights similarities with the mechanism for pore formation of the group of β-PFTs, despite these two groups having quite distinct architectures at the transmembrane region.

## Author Contributions

K. M., J. M. G. M., and J. M. M. C. conceived the study. K. M., J. M. G. M., K. T., and J. M. M. C. coordinated the study. K. M. performed the monolayer, leakage, and protein susceptibility assays. K. M., A. B., D. G. C., and M. V. carried out the cryo-EM study. S. S. and L. R. M. performed and analyzed the AFM experiments. A. B. carried out initial DNA cloning experiments. K. M., A. B., and J. S. performed imaging experiments with GUVs. K. M. and J. M. M. C. wrote the paper with input from all other authors. All authors approved the manuscript.

## Footnotes

5 The abbreviations used are:

PFT: pore-forming toxin
FraC: fragaceatoxin C
SM: sphingomyelin
DLPC: 1,2-dilauroyl-*sn*-glycero-3-phosphocholine
DPPC: 1,2-dipalmitoyl-*sn*-glycero-3-phosphocholine
DOPC: 1,2-dioleoyl-*sn*-glycero-3-phosphocholine
DiIC18: 1,1′-dioctadecyl-3,3,3′,3′-tetramethylindocarbocyanine perchlorate
LUV: large unilamellar vesicle
GUV: giant unilamellar vesicle
AFM: atomic force microscopy
cryo-EM: single-particle cryo-electron microscopy
PK: proteinase K
ANTS: aminonaphthalene-1,3,6-trisulfonic acid
PC: phosphatidylcholine.
